# Drug repurposing for next-generation combination therapies against multidrug-resistant bacteria

**DOI:** 10.7150/thno.56205

**Published:** 2021-03-04

**Authors:** Yuan Liu, Ziwen Tong, Jingru Shi, Ruichao Li, Mathew Upton, Zhiqiang Wang

**Affiliations:** 1 College of Veterinary Medicine, Yangzhou University, Yangzhou, Jiangsu, China.; 2 Jiangsu Co-innovation Center for Prevention and Control of Important Animal Infectious Diseases and Zoonoses, Joint International Research Laboratory of Agriculture and Agri-Product Safety, the Ministry of Education of China, Yangzhou University, Yangzhou, Jiangsu, China.; 3 Institute of Comparative Medicine, Yangzhou University, Yangzhou, Jiangsu, China.; 4 School of Biomedical Sciences, University of Plymouth, Drake Circus, Plymouth, UK.

**Keywords:** antimicrobial resistance, antibiotic adjuvants, combination therapies, drug repurposing, multidrug-resistant bacteria

## Abstract

Antimicrobial resistance has been a global health challenge that threatens our ability to control and treat life-threatening bacterial infections. Despite ongoing efforts to identify new drugs or alternatives to antibiotics, no new classes of antibiotic or their alternatives have been clinically approved in the last three decades. A combination of antibiotics and non-antibiotic compounds that could inhibit bacterial resistance determinants or enhance antibiotic activity offers a sustainable and effective strategy to confront multidrug-resistant bacteria. In this review, we provide a brief overview of the co-evolution of antibiotic discovery and the development of bacterial resistance. We summarize drug-drug interactions and uncover the art of repurposing non-antibiotic drugs as potential antibiotic adjuvants, including discussing classification and mechanisms of action, as well as reporting novel screening platforms. A pathogen-by-pathogen approach is then proposed to highlight the critical value of drug repurposing and its therapeutic potential. Finally, general advantages, challenges and development trends of drug combination strategy are discussed.

## Introduction

Sulfonamides, a group of synthetic antibiotics discovered in the 1930s, were the first chemical substances systematically used to treat and prevent bacterial infections in humans 1. However, their use has been overshadowed by the discovery of more efficacious and safer natural antibiotics, such as penicillin in 1929 2 and streptomycin in 1943 3. These unprecedented discoveries opened the Golden Age of antibiotics. It is undoubted that the successful introduction of antibiotics into clinical practice underpinned the development of modern medicine 4. Although antibiotics are essential in controlling infectious diseases in humans and animals 5, their applications have been widely abused (overused or misused), in particularly as growth promoters of animals in livestock breeding 6. Besides, the gains from antibiotic discovery are rapidly counteracted by the emergence of antibiotic resistance 7, 8. The emergence of drug resistance is currently explained by two propositions. First, the congenital theory suggests that antibiotic resistance is ancient 9, 10. In this theory, antibiotic resistance is believed to be naturally occurring prehistorically, including in antibiotic-producing organisms that have existed for millennia. For example, the complete vancomycin resistance determinant VanA was detected from 30,000-year-old Beringian permafrost sediments, which further supports this theory 9. A second theory proposes that antibiotic resistance is an acquired biological phenomenon because of the frequent use and overuse of antibiotics in the clinical setting and in agricultural processes 11, 12. According to this latter theory, the use of antibiotics creates a selective pressure, driving the emergence of bacterial resistance. Despite these debates, antimicrobial resistance (AMR) has now developed into one of the greatest challenges to public health worldwide 13.

The growing public health challenge caused by AMR necessitates novel regimens and approaches. Since the mid-1960s, the identification of new and effective antibiotic scaffolds using the traditional Waksman platform approach was challenging 14, 15. Actually, most specialized metabolites had considerable pharmacological or toxicological drawbacks because they were not initially designed as drugs. Additionally, given the fact that the generation of resistant bacteria by the horizontal transfer of resistance genes between bacteria or chromosomal mutation takes an average of 2 years, thus the development rate of antibacterial drugs is far behind the speed of bacterial resistance. Consequently, there is a growing gap between the clinical need for new antibiotics and new drug discovery and development. Because of the existing scientific and commercial challenges in drug development, it is increasingly challenging to find new antibiotics for clinical application.

Given these obstacles coupled with a very low output-to-input ratio, the majority of pharmaceutical industries have systematically dismantled antibiotic-discovery programs and shed expertise in antibiotic drug development in the past two decades 16. Therefore, only a handful of new classes of antibiotics such as daptomycin 17 have been approved by the Food and Drug Administration (FDA) in recent decades. Although governments, non-profit and public health organizations have recently proposed some incentive schemes such as increased investment to arouse enthusiasm for the development of novel antibiotics, there has been very limited success. In addition, some alternatives to antibiotics such as anti-virulence agents, antibodies, probiotics and vaccines have been explored as novel alternative therapies 18-22. However, these alternatives will probably best serve as adjunctive or preventive therapies in clinical practice as their effectiveness and safety as a monotherapy is not guaranteed. In this case, conventional antibiotic treatments are still indispensable.

In light of this, development of novel therapeutic strategies to combat drug-resistant pathogens is imperative. Accordingly, combination therapies provide promising therapeutic avenues to bypass the huge investment in the development of new drugs 23. An ideal drug combination should simultaneously meet the following three objectives: (1) has a synergistic effect that improves drug efficacy, (2) suppresses the emergence of spontaneous resistance and (3) attenuates drug toxicity to the host cells. In particular, repurposing non-antibiotic drugs (also known as antibiotic adjuvants) that have undergone extensive toxicological and pharmacological analysis is an effective method to reduce the time, cost and risks associated with conventional antibiotic innovation 24-26.

In this review, we outline the current knowledge on the molecular mechanisms of antimicrobial resistance in bacterial pathogens, which are pivotal in identifying effective drug combinations. Subsequently, we describe drug combination strategies that cover all typical classifications and their mechanisms of action. We focus particularly on repurposing non-antibiotic drugs as novel antibiotic adjuvants and discuss their merits in the development of next-generation combinational therapies. Furthermore, we summarize recent successes of antibiotic adjuvants in combating clinically important drug-resistant pathogens, including methicillin-resistant *Staphylococcus aureus* (MRSA), carbapenem-resistant Enterobacterales (CRE), MCR-producing Enterobacterales (MCRPE) and Tet(X)-expressing bacteria. Finally, the challenges of these combinations and future development for broad-spectrum combination therapies are highlighted.

## Molecular mechanisms of antimicrobial resistance

To counteract the action of antibiotics, bacteria have evolved versatile strategies, including intrinsic and acquired resistance 27. Intrinsic resistance to some specific antibiotics commonly occurs in certain bacterial species due to the presence of inherent structural or functional characteristics. For example, glycopeptide vancomycin 28 and rifampicin 29 are strongly active against Gram-positive bacteria, but ineffective against Gram-negative bacteria. This is predominantly due to an intrinsic difference in the composition of the cell envelop between Gram-positive and Gram-negative bacteria 30. The highly impermeable outer membrane (OM) of Gram-negative organisms prevents the entry of antibiotics effective against Gram-positive bacteria 31. Using high-throughput screening of random genome mutant libraries, several key genes have been identified that account for the intrinsic resistance of bacteria to antibiotics 32, 33. For instance, the inactivation of non-essential *E. coli* genes identified putative targets, including thioredoxin (TrxA) and thioredoxin reductase (TrxB), can greatly promote the activity of rifampicin 33. Biofilms represent another form of antibiotic intrinsic resistance determinants. These are surface-attached groups of microbial cells encased in an extracellular matrix. Various molecular mechanisms are involved in biofilm-mediated resistance, including reduced growth rates and the interaction of antimicrobials with biofilm matrix components 34.

**Figure 1:**
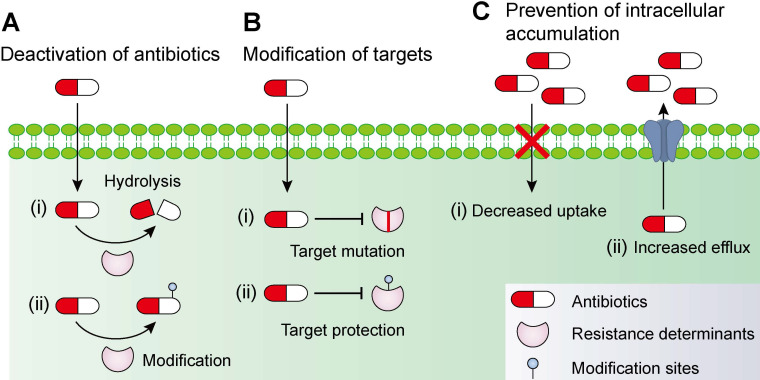
** Molecular mechanisms of antimicrobial resistance in bacterial pathogens.** Bacteria have evolved multifaceted strategies to counteract antibiotic killing, including **(A)** deactivation of antibiotics by hydrolysis or modification with resistance determinants, **(B)** modification of antibiotic targets by mutation or protection, and** (C)** prevention of intracellular accumulation of antibiotics by decreased uptake and increased activity of efflux pumps.

In addition to intrinsic resistance, bacteria could acquire or develop resistance to antibiotics by obtaining an exogenous resistance gene either via horizontal gene transfer mechanisms (e.g. conjugation, transformation and transduction) 35, 36 or chromosomal mutation 12, 37. Collectively, antibiotic resistance may be accomplished via three different mechanisms 27, 38, including inactivation of antibiotics by hydrolytic or chemical modification enzymes; modification of antibiotic targets; and prevention of intracellular accumulation of antibiotics owing to upregulation of active efflux and downregulation of OM permeability **(Figure 1)**. Understanding the genetic basis of bacterial resistance, coupled with the antibacterial spectrum of antibiotics, may guide the development of new combination therapies with improved or expanded activities against target pathogens.

### Deactivation of antibiotics

The enzyme-catalyzed deactivation of antibiotics is one of the important drivers that confers antibiotic resistance. Hitherto, thousands of resistance enzymes that can degrade or modify different classes of antibiotics, including β-lactams, aminoglycosides, phenicols and macrolides, have been identified 39. β-lactamase is a typical resistance enzyme, comprising of serine-β-lactamase and metallo-β-lactamase. Development of new β-lactamases evolves with the introduction of new β-lactams, which have been reviewed in previous reports 40-42. The early β-lactamases are only active against the first-generation β-lactams, followed by extended-spectrum β-lactamases (ESBLs) that have hydrolytic activity against oxyimino-cephalosporins. Alarmingly, the carriage of diverse ESBLs and carbapenemases 43, 44, including KPC and NDM enzymes in a range of Gram-negative organisms, has underpinned the emergence of isolates that are resistant to almost all β-lactam antibiotics.

The addition of chemical groups to the key active centers of antibiotics may also impair their activity by preventing antibiotics from binding to their targets partly due to steric hindrance. Various different chemical groups including acyl, phosphate, nucleotidyl and ribitoyl groups can be transferred by corresponding enzymes. One of classic examples is the aminoglycoside antibiotics, which possess numerous exposed hydroxyl and amide groups that are particularly susceptible to modification 45. Aminoglycoside-modifying enzymes (AMEs) comprise of three main classes: acetyltransferases, phosphotransferases and nucleotidyltransferases 46, 47. Worryingly, all three AMEs classes were identified in *Campylobacter coli* isolated from broiler chickens in China and these strains were resistant to almost all aminoglycoside antibiotics 48. The rif-associated-element (RAE) encoded phosphotransferase has also resulted in rifampicin resistance in Actinomycetes and other bacterial pathogens 49. Furthermore, tigecycline resistance genes *tet*(X) and its variants are responsible for flavin-dependent (FAD) monooxygenase, which selectively hydroxylates tigecycline to form 11a-hydroxytigecycline and decreases its affinity for 30S subunit of the bacterial ribosome 50, 51.

### Modification of targets

In addition to inactivating antibiotics, some bacteria attempt to remodel themselves to gain resistance to antibiotics. This is because most antibiotics mainly act through a single target. The protection of targets has been a clinically relevant mechanism of resistance for several important antibiotics. For example, chloramphenicol-florfenicol resistance (*cfr*) methyltransferase could specifically methylate A2503 in the 23S rRNA, thereby conferring resistance to five classes of antibiotics, including phenicols, pleuromutilins, streptogramins, lincosamides and oxazolidonones 52, 53. Importantly, the *cfr* gene is usually located on conjugative plasmids, which function as vectors facilitating their wide intra- and inter-species dissemination 54.

Colistin, a cyclic antimicrobial peptide with long and hydrophobic tails, is effective against Gram-negative bacteria through binding with the anionic lipopolysaccharide (LPS) component of the bacterial OM, leading to membrane destabilization 55. Due to a paucity of alternatives, colistin is recognized as one of the last-resorts of defense against MDR Gram-negative pathogens. As a consequence, bacterial resistance to colistin has developed. Initially, colistin resistance was limited to chromosomal changes such as *pmrA/pmrB* activation of *arnBCADTEF* and *pmrE*, which collectively modifies LPS by the addition of 4-amino-4-deoxy-L-arabinose 56. However, a novel mobilized colistin resistance gene (*mcr*) has been reported in bacteria of both animal and human sources 57. An *mcr*-encoded phosphoethanolamine transferase can add phosphoethanolamine to lipid A, hence, reducing colistin binding through the lowering of the negative charge of LPS.

### Prevention of intracellular accumulation

The access of antibiotics to their cellular targets has been proven to be critical for their antibacterial activity 58. Thus, some bacteria confer resistance to antibiotics by reducing membrane permeability or enhancing the activity of efflux pumps. Compared with Gram-positive species, Gram-negative bacteria are intrinsically less permeable to many antibiotics as their OM forms a permeability barrier. Nevertheless, hydrophilic antibiotics can cross the OM through the porin proteins. Downregulation of porins or the replacement of porins with more-selective channels would reduce the permeability of the OM, limiting antibiotics entry into the bacterial cells. For example, reduced porin production can also result in carbapenem resistance in non-carbapenemase-producing Enterobacterales 59.

Bacterial efflux pumps actively transport various antibiotics out of the cell and are major contributors to the resistance of Gram-negative pathogens to many clinically used drugs. The biochemistry and genetics of multidrug efflux pumps in a panel of Gram-negative organisms have been well-reviewed 60. Some efflux pumps have narrow substrate specificity (e.g., Tet pumps), but many carry a wide range of structurally different substrates and are called MDR efflux pumps. One of the most well-studied MDR efflux pumps is the resistance-nodulation-division (RND) pump 61, which is a clinically relevant efflux transporter with a wide range of substrates in almost all Gram-negative bacterial pathogens. RND pumps, such as AcrB in *E. coli* and enterobacteria, form a tripartite complex (AcrAB-TolC) with a periplasmic adaptor protein AcrA and an OM channel TolC 62. The first structure and biofunction of AcrAB-TolC, as well as the stoichiometry and key interactions between residues, have been elucidated using cryo-electron microscopy 63.

AcrAB-TolC plays a central role in phenotypic heterogeneity or plasmid-conferred drug-resistance acquisition. For instance, biased partitioning of the AcrAB-TolC was found to be an important driver that underlay the phenotypic variation in isogenic bacterial populations 64. Using live-cell microscopy, Nolivos and colleagues showed that the acquisition of tetracycline-efflux pump TetA in the presence of translation-inhibiting antibiotics was dependent on AcrAB-TolC, which reduced the accumulation of intracellular antibiotics and gained time for TetA expression 65. Worryingly, multiple genes that encode MDR efflux pumps have been mobilized from chromosomes to conjugative plasmids. For example, an IncH1 plasmid from a *Citrobacter freundii* strain carrying both RND efflux pump gene cluster and *bla*_NDM-1_ genes was found 66. Moreover, a plasmid-encoded RND pump carrying novel gene cluster *tmexCD1-toprJ1* was recently identified in *K. pneumoniae*, which conferred resistance to multiple drugs including tigecycline 67. These examples demonstrate the transmissibility of efflux pump-mediated antibiotic resistance among clinically relevant pathogens.

## Drug combinations strategy

### Drug-drug interactions

As discussed above, historic abuse of antibiotics may soon lead to an era of scarcity of effective antibiotic drugs. Because of this developing crisis, accelerated creation of new drugs or repositioning of the existing ones is going to be essential. To counteract surging AMR as well as promoting the availability of new antibiotic drugs, innovative combination strategies to produce more viable and potent drugs from the current arsenal are warranted.

**Figure 2:**
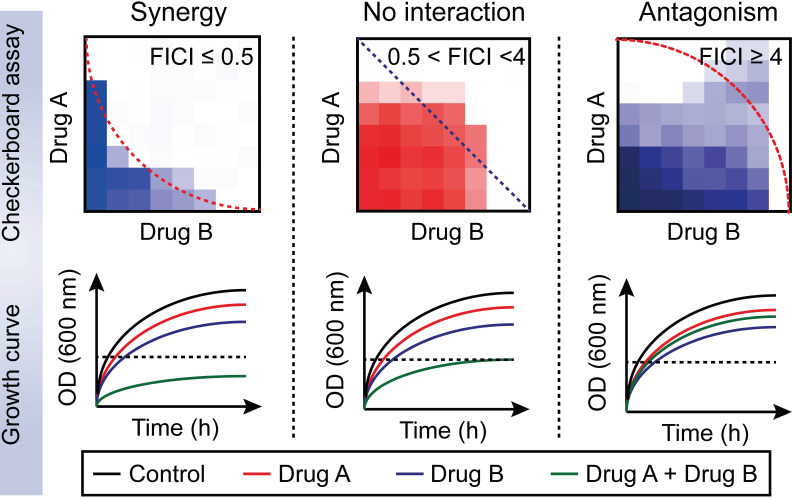
** Distinguishing between three drug-drug interactions, including synergy, no interaction and antagonism.** Drug-drug interactions can be evaluated by using a checkerboard assay with determination of the fractional inhibitory concentration index (FICI) or bacterial growth curves in the combination of sub-lethal (one-quarter minimal inhibitory concentration (MIC)) concentrations of drug A and B. Synergy is defined as an FICI of ≤0.5 and a significantly reduced bacterial growth curve in the presence of paired drugs. No interaction, including additive and indifference, is defined as an FICI of >0.5 and <4. Antagonism is defined as an FICI of ≥4 and an enhanced bacterial growth curve compared with monotreatment.

Drug-drug interactions can be divided into three types: synergy; no interaction; and antagonism 68. Furthermore, no interaction includes additive and indifferent effects. Drug-drug interaction can be determined in the microbiology laboratory using the fractional inhibitory concentration index (FICI). This approach is achieved with the checkerboard assay **(Figure 2)** 69. The Fractional inhibitory concentration (FIC) is defined as the minimum inhibitory concentration (MIC) of compound A in the presence of B divided by the MIC of A, while the FICI is the sum of FIC_A_ and FIC_B_ based on the following formula:


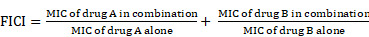


The drug combination is defined to be synergistic with an FICI value ≤0.5 and suppressed bacterial growth in the combination group; additive with 0.5< FICI≤1; indifferent with 1< FICI <4; and antagonistic with FICI≥4 and enhanced bacterial growth curves in the combination treatment 68.

### Classification and mechanisms of synergistic or additive combinations

**Figure 3:**
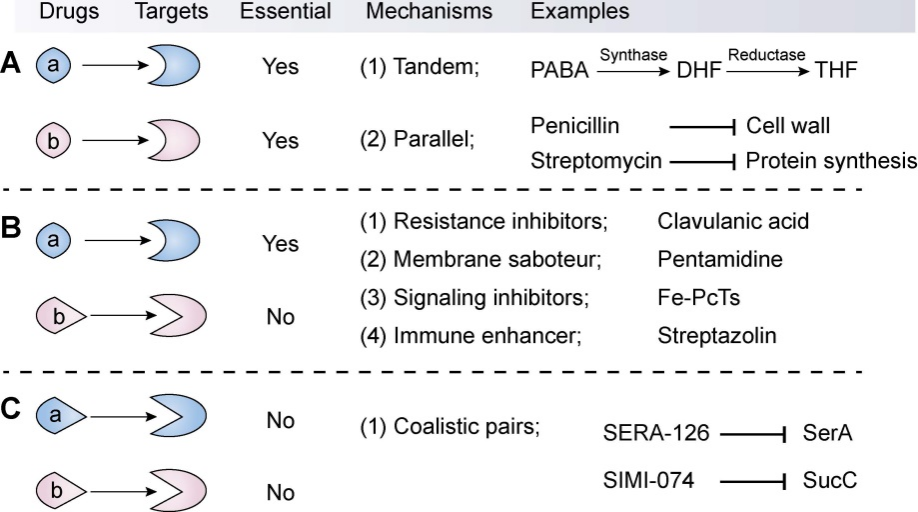
** Classification, mechanisms and examples of synergistic combination in the fight against bacterial pathogens.** The synergistic combinations can be divided into three types according to their modes of action. The first type **(A)** is the synergistic combination of two antibiotics (a and b), which target distinct essential molecular processes by way of a tandem or parallel manner. The second type **(B)** combination comprises an antibiotic (a) that targets an essential process and a non-antibiotic adjuvant (b) that suppresses resistance determinants or non-essential processes or enhances the host immune response. In particular, this type displays great potential in the development of novel antibiotic adjuvants. In contrast, the third combination **(C)** refers to two non-antibiotic agents that target non-essential but synthetically lethal gene functions.

The available synergistic or additive drug combinations are commonly classified into three classes 70. First is the combination of an antibiotic with another antibiotic **(Combination I, Figure 3A)**. The two antibiotics act synergistically by targeting distinct essential molecular processes in a tandem or parallel manner. For example, sulfonamides and trimethoprim (its potentiator) can competitively bind to dihydrofolate synthase and reductase, respectively, thereby blocking the biosynthesis of tetrahydrofolate. Tetrahydrofolate, a coenzyme of one-carbon unit transferase, is involved in the synthesis of the nucleic acid precursors such as purine and pyrimidine 71. By contrast, the combination of penicillin with streptomycin displays superior efficacy over individual agents via targeting unrelated targets: synthesis of the cell wall and protein. These classic antibiotic combinations have been proved effective both clinically and epidemiologically 72, thus they are still clinically used as first-line drugs. However, through redundant mechanisms in the same organisms, bacterial pathogens are increasingly becoming resistant to all available antibiotic drugs 73. Therefore, the efficacy of antibiotic combinations is gradually becoming diminished.

The second combination comprises of an antibiotic and a non-antibiotic agent that has no direct antibacterial activity **(Combination II, Figure 3B)**. This kind of combination is the major focus of this review. Non-antibiotic compounds with little or no antibiotic activity but which unexpectedly enhance the efficacy of antibiotics are termed as antibiotic adjuvants or potentiators. Based on the modes of action, antibiotic adjuvants can be divided into four classes: (i) resistance inhibitors; (ii) membrane saboteurs; (iii) signaling inhibitors; and (iv) immune enhancers 74-76.

### Resistance inhibitors

Class I adjuvants target bacterial resistance enzymes and efflux pumps. As previously described, resistance enzymes impair antibiotic activity through multiple pathways, such as antibiotic hydrolysis or modification, or modification of antibiotic targets. Inhibition of enzyme-mediated drug resistance has been proven to be a clinically successful regimen to restore the activity of specific antibiotics 77. The most successful examples are β-lactamase inhibitors (as reviewed in Ref. 78, 79).

β-lactamases hydrolyze the β-lactam ring of the antibiotics, which is essential to their antimicrobial activity, through two distinct chemical mechanisms 80. The first mechanism uses an active-site Ser residue that forms a transient covalent bond with the antibiotic followed by hydrolysis of the enzyme-associated ester to generate the inactive antibiotics, while the second mechanism achieves β-lactam hydrolysis through metal (usually two Zn^2+^ ions)-assisted activation of a water molecule to generate the hydrolytic species. Serine-β-lactamases such as TEM, SHV and CTX-M have historically been the dominant enzymes in bacterial pathogens, but in the past few years, metallo-β-lactamases (for example, NDM and VIM) have been increasingly problematic in clinical practices. The unprecedented discovery of clavulanic acid as β-lactamase inhibitors led to the first antibiotic-adjuvant combinations (amoxicillin-clavulanic acid pair, also called Augmentin) 81 and the more recently FDA-approved avibactam (a new class of serine-β-lactamase inhibitor) in 2016 and vaborbactam in 2017. The combinations of avibactam with ceftazidime (avycaz) and vaborbactam with meropenem (vabomere) have been successfully introduced into the market.

The efflux pump inhibitors are also important resistance inhibitors. Energy-dependent drug efflux mechanisms are ubiquitous in various bacteria and are critical in mediating clinical antibiotic resistance. Among the numerous families of transporters, several contain prominent members of efflux transporters. RND, MFS (major facilitator superfamily), MATE (multidrug and toxic compound extrusion), SMR (small multidrug resistance), and ABC (ATP-binding cassette) superfamilies or families are particularly important in bacteria 82. Remarkable scientific and technological advances have allowed for an in-depth understanding of their structural and biochemical basis, substrate profiles and molecular regulation, which contribute to the discovery of novel efflux pump inhibitors. Systematic summaries of efflux pump inhibitors have been previously reviewed 83, 84. Despite these ongoing efforts, no efflux pump inhibitor has been clinically approved for therapeutic use.

### Membrane saboteurs

Antibacterial activity of majority effective antibiotics with intracellular targets is highly dependent on a sufficient intracellular level of the drugs. Hydrophobic antibiotics diffuse through the lipid bilayer, whereas hydrophilic antibiotics enter only through bacterial porins. Thus, membrane composition, membrane lipids and porins all influence membrane permeability and affect the susceptibility of bacteria to antibiotics. As described in **Section 2**, additional OM in Gram-negative organisms compared with Gram-positive bacteria or overexpression of porins confers resistance to the antibiotic 85. Therefore, enhanced membrane permeability or drug uptake by antibiotic adjuvants would potentiate the activity of some specific antibiotics against drug-resistant bacteria. For example, various Gram-positive active antibiotics such as rifampicin would be effective against Gram-negative bacteria when in combination with membrane saboteurs such as detergents, surfactants and antimicrobial peptides 86. Specifically, surfactant glycerol monolaurate (GLM) and cationic α-helical antimicrobial peptide mastoparan-C analogs were found to synergize with gentamicin or rifampicin, respectively 87, 88. Meanwhile, a series of repurposed compounds such as oxyclozanide and toremifene displayed direct antibacterial activity against bacteria via membrane damage 89, but the potentiating activity of these membrane saboteurs are still unknown.

Furthermore, some compounds have been found to increase the uptake of antibiotics, thereby overcoming intrinsic resistance. For example, loperamide, an antidiarrheal drug, was identified as an adjuvant of the semi-synthetic tetracycline antibiotic minocycline 90. Additional experiments showed that loperamide decreased the electrical component (Δψ) of proton motive force (PMF). To counter this effect and maintain ATP synthesis levels, bacteria increase the pH gradient (ΔpH) across the inner membrane, which in turn facilitates the uptake of tetracycline antibiotics. This result was consistent with previous notion that uptake of tetracyclines is dependent upon ΔpH 91, whereas aminoglycoside uptake is driven largely by Δψ 92. These findings suggest the screening of additional compounds that perturb Δψ or ΔpH in bacteria may lead to the identification of tetracycline or aminoglycoside adjuvants, respectively. Interestingly, a series of tobramycin-based hybrids have been designed and shown to potentiate legacy antibiotics against multiple pathogens through permeabilizing the OM and dissipation of the PMF across the inner membrane 93. For instance, the amphiphilic tobramycin-lysine conjugates can also reduce the Δψ component of PMF and potentiate the activity of rifampicin and minocycline against MDR Gram-negative pathogens including *P. aeruginosa* 94. Non-canonical tobramycin-based antibiotic adjuvants such as tobramycin-cyclam conjugates potentiated β-lactam antibiotics and β-lactam antibiotic/β-lactamase inhibitor combinations against carbapenem-resistant *Pseudomonas aeruginosa* 95-97. In addition, the combination of membrane saboteurs and efflux pump inhibitors may strength the synergistic effect on antibiotics. For instance, the conjugation of tobramycin to efflux pump inhibitors was proven to enhance the activity of tetracycline antibiotics through permeating the OM and resisting efflux 98.

### Signaling inhibitors

Bacteria perceive changes in the external environment through a two-component system (TCS) comprising of a sensor kinase and a response regulator 99. Interestingly, bacteria have employed distinct signal transduction systems to respond to various environmental changes, including changes in the pH, level of nutrients and the presence of antibiotics. Thus, some TCS pathways play a critical role in mediating antibiotic resistance. For example, in Gram-negative bacteria, it has been suggested that AmgRS, PhoPQ and VbrKR can lead to aminoglycoside, polymyxins and β-lactams resistance, respectively 100, 101. Inactivation of AmgRS also enhanced tobramycin activity (8 to 16-fold) against *P. aeruginosa* 102. Additionally, AmgRS inactivation resulted in reduced virulence and improved eradication of mature biofilm. These results demonstrate that TCS such as AmgRS may be a potential antibiotic adjuvant target to combat *P. aeruginosa*. An overview of small-molecule inhibitors of TCS, including the tyramine derivative RWJ-49815 and closantel, has been systemically reviewed 103. Nevertheless, the potentiating effect of these TCS inhibitors on antibiotics warrants more exploration.

Besides, another important signaling system, RecA, is also involved in multiple biological processes such as DNA repair, SOS response, biofilm formation and evolution of resistance 104. By contrast, inhibition of RecA by exogenous compounds such as iron(III) phthalocyanine tetrasulfonic acid (Fe-PcTs) prevented SOS response pathway activity and acquisition of resistance by mutation or horizontal gene transfer, thus enhancing the killing by bactericidal antibiotics 105. These observations indicate that inhibitors of bacterial signaling might serve as potential antibiotic adjuvants.

### Immune enhancers

Given the critical role of the host defense mechanism in confronting invasive bacteria, enhancing host defense mechanisms offers an alternative set of targets for antibiotic adjuvants. For example, streptazolin, a natural product, was found to enhance macrophage activity against *Streptococcus mutans* through the upregulation of nuclear factor-kappa B (NF-kB) 106. Generally, an immune enhancer provides a universal potentiation for almost all antibiotic treatments. However, over-activation of the immune system would produce deleterious effects on the host.

The third combination comprises of two non-antibiotic compounds that target non-essential but synthetically lethal gene functions **(Combination III, Figure 3C)**. For instance, Aziz *et al.* performed virtual and biological screens, leading to the discovery of several synthetic lethal pairs in Gram-negative bacteria, such as the combination of SERA-126 and SIMI-074 by targeting SerA (phosphoglycerate dehydrogenase) and SucC (succinyl-CoA synthetase), respectively 107. Notably, this combination strategy may minimize the emergence of resistance because these compounds possess poor antibacterial activity and thus afford a weak drug-selection pressure on microbial populations. Nevertheless, the emergence of resistance to one drug will be enough to invalidate this kind of synergistic combination that act by targeting two non-essential but synthetically lethal gene products in bacterial pathogens 108.

## Screening approaches for identification of effective drug combinations

### Traditional screening approaches

The checkerboard assay has been one of the traditional screening approaches employed in the search for synergistic or additive drug combinations 109. As described above, the FICI, which is determined by the checkerboard assay, is a critical indicator to infer drug-drug interactions. In addition, the bacterial growth profile in the presence of sub-lethal concentrations of two compounds alone or their combination is also a simple means to achieve preliminary screening. Frequently, follow-up time-kill studies are utilized to confirm synergism. In these studies, a reduction of colony-forming units (CFU) with a factor of at least 2log10 per milliliter compared to monotreatment was further used to define a synergistic combination. Together, these screening approaches are based on bacterial growth changes under mono and combinational treatment. Traditional screening approaches are advantageous due to its simplicity and high operability under limited experimental conditions. Nevertheless, these techniques are crude measures of synergy and may conceal more subtle drug combination pairs. In addition, there methods are also low-throughput screening approaches that are not suitable for large-scale screening.

### New screening approaches

**Figure 4:**
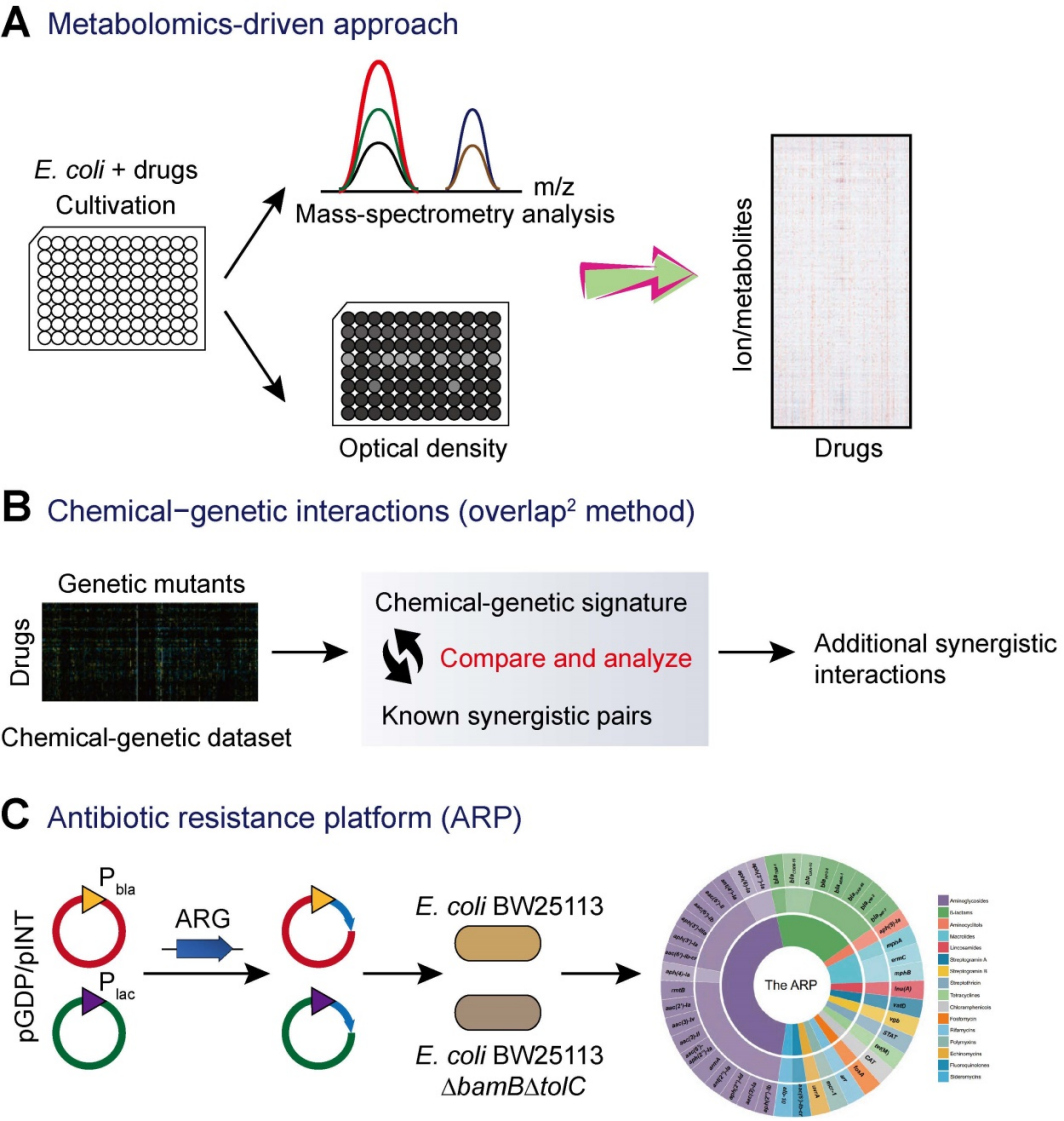
** Novel screening approaches for antibiotic adjuvant discovery. (A)** Metabolomics-driven approach to predict novel combination antimicrobial therapies 110. Metabolic profiling of *E. coli* after 2 h of drugs treatment were analyzed by flow injection analysis in a time of flight mass spectrometer (FIA-TOFMS), while bacterial growth in the presence of drug was monitored using a plate reader over 6 h. **(B)** Exploring additional synergistic interactions on the basis of chemical-genetic interactions analysis 111, 112. **(C)** The antibiotic resistance platform (ARP) allows for the discovery of new antibiotic adjuvants 113. The platform consists of a cell-based library of *E. coli* expressing individual resistance genes. The expression of resistance genes is regulated by the utilization of two series of plasmids and two different promoters (strong P_bla_ promoter and the weaker P_lac_ promoter). Subsequently, these constructs were transformed into wild-type *E. coli* and/or the hyperpermeable efflux-deficient mutant *E. coli* BW25113 *△bamB△tolC.*

In addition to the traditional screening approaches based on bacterial growth, technical advance in sequencing and random transposon mutagenesis have fostered the development of new screening techniques for the discovery of novel antibiotic adjuvants from huge libraries of non-antibiotic agents. Recently, a combined experimental-computational approach based on high-throughput metabolomics was developed to predict drug-drug interactions **(Figure 4A)** 110. This study applied high-throughput metabolomics to monitor the metabolic response of *E. coli* to a library of 1,279 chemical compounds (the Prestwick Library), most of which are human-targeted drugs that have little or no direct antimicrobial activity. By combining the newly generated drug metabolome profiles with metabolic and fitness profiles in *E. coli* gene-knockout mutants, the authors made *de novo* predictions of modes of action of drugs and systematically predicted epistatic drug-drug interactions. As a result, several novel drug combinations including sulfamethizole and zidovudine were identified for the first time 110.

Another promising approach is the exploration of chemical-genetic interactions for the identification of novel synergistic small-molecule pairs. A representative example is termed the overlap^2^ method (O2M) 111. This method applied known synergistic interactions to predict many additional interactions based on large-scale chemical-genetic data **(Figure 4B)**. First, a collection of mutants is grown in the presence of different small molecules to generate a chemical-genetic dataset. Then, a quantitative growth score and a chemical-genetic signature for each combination of mutant and molecule were calculated based on the colony size. The mutants that showed significant growth scores to known synergistic pairs could be used as synergy prediction mutants. Thus, other compounds that induced significant phenotypes in this mutant would synergize with the corresponding antibiotics. Subsequently, the synergistic activity was further validated by checkerboard analysis. This method has been successfully applied in the screening of synergistic small-molecule pairs for combating antibiotic-resistant bacteria. A library of 2,000 small molecules was screened, and several molecules such as azidothymidine were found to synergize with trimethoprim and/or sulfamethazine against resistant clinical *E. coli* and *K. pneumoniae* 112.

In addition, a recent study described a new platform termed “antibiotic resistance platform” (ARP) where individual resistance elements are cloned into a uniform *E. coli* host under the control of constitutive strong (*bla*) and weak (*lac*) promoters **(Figure 4C)** 113. Consequently, an array of transformed *E. coli* that could express one of greater than 40 different known antibiotic resistance genes (ARGs) was constructed. This platform presents a streamlined screening and testing tool that can improve traditional screening of drug-resistant pathogens, which often have poorly characterized genotypes and redundant resistance elements. Most importantly, this allows for the identification of new compounds that target the resistance genes or their products, thus rejuvenating the therapeutic potentials of existing antibiotics. However, considering that this platform is designed by in engineered* E. coli* with fully sequenced genetic background, it is not possible to determine whether the screened compounds would be equally effective against clinical drug-resistant strains.

## Recent successes in the fight against superbugs

### Methicillin-resistant *Staphylococcus aureus* (MRSA)

*Staphylococcus aureus* is one of the most important opportunistic pathogens and causes a range of hospital and community-acquired infections 114. It can invade a variety of tissues and has a collection of virulence factors, which provide protection from the host immune system and results in many toxin-mediated diseases such as toxic shock syndrome 115. Notably, *S. aureus* is initially susceptible to clinically relevant antibiotics. Clinical application of semisynthetic antibiotics like methicillin in the 1950s rapidly led to the emergence of methicillin-resistant *S. aureus* (MRSA) in the 1960s 116. MRSA isolates can produce an altered penicillin-binding protein (encoded by an acquired *mecA* gene), which displays a weak affinity for methicillin type antibiotics 117. Infections caused by MRSA results in higher mortality, as well as increased treatment time and health care costs 118. Alarmingly, the increased use of vancomycin, last resort for the treatment of MRSA-associated infections, has already led to the emergence of vancomycin-intermediate *S. aureus* (VISA) and vancomycin-resistant *S. aureus* (VRSA) 119, although numbers of infections with VRSA reported clinically remain low. Repurposing previously approved drugs as potential antibiotic adjuvants offers a feasible approach against MRSA **(Table 1)**.

Recently, Esther et al. found that membrane-carotenoid interaction with the scaffold protein flotillin led to functional membrane microdomains (FMMs) forming in MRSA 120. FMMs facilitated efficient oligomerization of multimeric protein complexes that involve PBP2a, which was responsible for penicillin resistance in MRSA. Interestingly, lipid-lowering drugs such as zaragozic acid disrupted FMM assembly, thereby interfering with PBP2a oligomerization and reversing MRSA penicillin resistance both *in vitro* and *in vivo* 120. In addition, using a cell-based screen of about 45,000 diverse compounds, Omar et al. discovered a potent anti-virulence agent named MAC-545496 that targets GraR (glycopeptide resistance-associated protein R, also known as antimicrobial-peptide sensor protein R), which reversed β-lactam resistance in the community-acquired MRSA USA300 strain 121. Staphylococcal accessory regulator A (SarA), a global virulence regulator, plays a critical role in pathogenesis and β-lactam antibiotic resistance in *S. aureus* 122-124. Interestingly, antidepressant and antiviral hypericin was found to significantly reduce *sarA*, *mecA* and virulence-related regulator expression, thereby enhancing the activity of β-lactam antibiotics against MRSA 125.

In another study, Farha et al. found that deletion of the wall teichoic acid (WTA) synthesis gene (*tarO*) resulted in enhanced sensitivity of MRSA to β-lactam antibiotics that target penicillin-binding protein (PBP) 126, suggesting the critical role of WTAs in the β-lactam resistance of MRSA. Based on this result, a screen of WTAs inhibitors was performed. Interestingly, ticlopidine, an antiplatelet drug was identified to strongly potentiate cefuroxime *in vitro* and in a *Galleria mellonella* infection model 126. This study provides evidence that WTA biogenesis represents an Achilles heel in the cooperative function of PBP, thereby providing a novel target in the discovery of β-lactams adjuvants to tackle MRSA infection. Screens for synergy between compounds have been widely used to reveal functional connections among cellular components. The utility of antagonism, however, has largely been overlooked. In one such study, Farha et al. conducted a high-throughput chemical screen for the antagonist of targocil and ticlopidine, inhibitors of WTA flippase in *S. aureus*. Using this approach, clomiphene, a widely used fertility drug, was identified as an inhibitor of undecaprenyl diphosphate synthase, an enzyme that catalyzes the synthesis of a polyisoprenoid essential for both peptidoglycan and WTA synthesis 127. Notably, clomiphene displayed synergistic activity with cell wall-targeted antibiotics against MRSA. Furthermore, a thioredoxin reductase (TrxR) inhibitor auranofin in combination with linezolid or fosfomycin showed synergistic antimicrobial activities against methicillin-sensitive *S. aureus* (MSSA) and MRSA both *in vitro* and *in vivo* 128. Ebselen, an antioxidant compound, also potentiated the activity of topical antimicrobials (mupirocin, fusidic acid, retapamulin and daptomycin) against MRSA 129.

### Carbapenem-resistant Enterobacterales (CRE)

Carbapenems are atypical β-lactam antibiotics with broad-spectrum and strong antibacterial activity. They exert their activity via binding to PBP and thereby inhibit cell wall synthesis 130. Carbapenems are preferred as the last option drugs for treatment of MDR bacterial infections 131. Enterobacterales are a family of diverse Gammaproteobacteria which include common (e.g. *Klebsiella pneumoniae* and *Salmonella enterica*) and rare (e.g. *Proteus mirabilis*) human pathogens with high resistance to carbapenems. According to the CDC report in 2019 (https://www.cdc.gov/drugresistance/pdf/threats-report/2019-ar-threats-report-508.pdf), carbapenem-resistant Enterobacterales (CRE) is listed as a top three key AMR threat. This is due to its rapidly increasing global spread, propensity for multidrug resistance, and high mortality during blood stream infections 44, 132.

Bacterial resistance to carbapenems mainly results from the hydrolysis of the β-lactam ring by dedicated carbapenemase enzymes. Zn(II)-dependent Metallo-β-lactamases (MBLs) such as NDM, VIM, IMP, and OXA-48 are the most common carbapenemases in CRE. Since the first identification in 2009 133, NDM-1-expressing bacteria have spread globally in over 70 countries due to the highly transferable of *bla*_NDM-1_-bearing plasmid and these strains cause various types of infections. Worryingly, there are few therapeutic regimens for CRE-associated infections 134.

A combination of carbapenems and MBLs inhibitors offers a more economical and effective way to counter CRE **(Table 2)**. Compared with the development of unknown compounds, repurposing previously approved compounds as potential MBL inhibitors highly shortens development time and cost, while ensuring the safety of drugs. Normally, MBL inhibitors have two different modes of action: sequestration of metal or formation of covalent bonds 135. An example of the metal ion binding is Zn-dependent inhibition of MBLs. This strategy depends on the fact that Zn^2+^ ions located active sites are essential for the catalysis of MBLs. Consistently, various chelators of metals such as EDTA have anti-MBLs activity. In 2014, a microbial natural product from a strain of *Aspergillus versicolor* known as aspergillomarasmine A (AMA) was identified as a potent inactivator of VIM-2 and NDM-1 through the removal of Zn^2+^ *in vitro* 136. This molecule has been confirmed as an inhibitor of angiotensin-converting enzyme (ACE) in the 1980s 137 and an endothelin-converting enzyme in the 1990s 138. AMA markedly restored meropenem activity in a mouse infection model 136. The carboxylate groups and the Asp residue in AMA were revealed to be important in its effective inhibitory effect on MBLs 139. Interestingly, another ACE inhibitors for modulating blood pressure such as captopril and its stereoisomers have also been found as the potential inhibitors of several MBL enzymes such as NDM-1 and VIM-1. This is because they can chelate zinc ions in the active site by a free thiol 140. Similarly, additional thiol-containing conventional approved drugs 141, 142 such as captopril (angiotensin-converting enzyme inhibitor) 140, disulfiram (alcohol-abuse drug) 143, thiorphan (enkephalinase inhibitor) 144 and tiopronin (miscellaneous genitourinary tract agent) 144 also exerted inhibitory activity on MBLs. In addition, benzene ring-rich compounds such as benzophenone 145 can also chelate metal ions of MBLs, thus suppressing the enzymatic activity.

The other mode of action of MBL inhibition is covalent bond formation, a Zn-independent inhibition of MBLs. For instance, cefaclor was identified as a covalent irreversible inhibitor of NDM-1 with *K*i of 2.3 ± 0.1 mM through multiple pathways. It is partially mediated by Lys211 146, thus providing a handhold for developing covalent NDM-1 inhibitors. In addition, the voltage-dependent calcium channel (VDCC) inhibitor ebselen was identified as a dual covalent inhibitor of MBLs 147. Ebselen can simultaneously bind to two sites in NDM-1 by forming an S-Se bond with Cys221 and an amide bond with Lys224, thus selectively inhibiting MBLs both *in vitro* and *in vivo*. Another inhibitor of MBLs is pterostilbene 148, a polyphenol compound that was isolated from red sandalwood. It has been used as a natural dietary antioxidant and in other applications such as anticancer. According to the results of enzyme inhibition assays, pterostilbene significantly inhibited NDM-1 hydrolysis activity on meropenem. Molecular dynamic simulation revealed that pterostilbene is localized to the catalytic pocket of NDM-1 by forming a strong interaction with Trp93 and Asp124. Consequently, this hinders substrate binding to NDM-1 and reduces NDM-1 activity 148. Furthermore, a high-throughput assay revealed that the inhibitor of topoisomerase II mitoxantrone effectively suppressed enzymatic activity of VIM 149, through unknown mechanisms.

### MCR-producing Enterobacterales (MCRPE)

**Figure 5:**
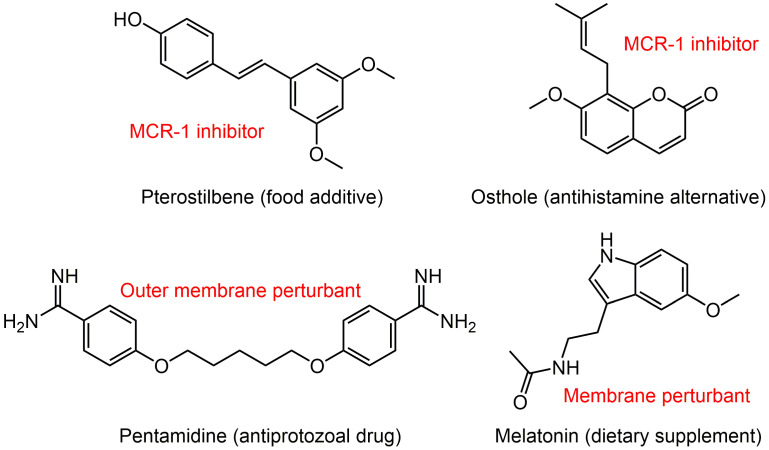
** Potential colistin adjuvants against MCR-producing Enterobacterales.** Four non-antimicrobial agents including food additive pterostilbene, antihistamine alternative osthole, antiprotozoal drug pentamidine and dietary supplement melatonin were found to potentiate colistin activity against MCR-producing Enterobacterales. The major mechanisms of action of these adjuvants in combination with colistin were presented next to the compounds in red font.

Colistin resistance has been reported since 2015 via a plasmid-mediated mobile gene *mcr-1* 57, which is problematic because it can be easily transmitted between different species and ecosystems (humans, animal and environment). More recently, a series of *mcr-1* variants including *mcr*-2 to *mcr*-10 have been identified in various species 150. Given that colistin is one of the last options against MDR bacterial infections, the European Medicines Agency updated the risk level of colistin resistance from low to high in 2016 151. To avoid the potential problems owing to the increase of the *mcr*-mediated resistance threshold, there is growing interest in effective combinations to combat MCRPE **(Figure 5)**.

A broth microdilution checkerboard method was applied to assess the synergistic effect between 115 natural compounds and polymyxin B. Consequently, the food additive pterostilbene was identified as a novel MCR-1 inhibitor 152. The FICI is 0.156 or 0.188 against MCR-producing *E. coli* strains of both human and animal origin. Meanwhile, its therapeutic effect was confirmed in a mice model of MCRPEC infection. Another natural product, osthole from the dried root of medicinal plants also effectively improved colistin activity in both* in vitro* and *in vivo* experiments by inhibiting MCR-1 activity 153. Besides, the FDA-approved anthelmintic drugs niclosamide and salicylanilides were demonstrated to have a synergistic effect with colistin recently 154, 155, which could be used against both colistin-sensitive and colistin-resistant Gram-negative bacilli that harbor the *mcr-1* gene.

The difficulty in eradicating Gram-negative bacteria is primarily due to their highly impermeable OM, which serves as a barrier to many effective antibiotics. To identify non-lethal molecules that perturb the OM, Stokes et al. screened 1,440 previously approved drugs for suppression of vancomycin activity against *E. coli* at 15°C 156. Consequently, antiprotozoal drug pentamidine was identified to effectively interfere with the Gram-negative OM by targeting LPS. Pentamidine exerted synergy with Gram-positive active antibiotics such as rifampicin and novobiocin against MCRPE *in vitro* and *in vivo* 156. This study developed an unconventional screening platform for the screening of non-lethal OM active compounds that are potential adjuvants of Gram-positive active antibiotics. In addition, it uncovered the therapeutic potential of pentamidine for treatment of MCRPEC infections. Similarly, our study found that the combination of colistin and dietary supplement melatonin, which has been approved for treating sleep disturbances and circadian disorders, enhanced bacterial OM permeability, promoted oxidative damage and inhibited the activity of efflux pumps, thereby overcoming MCR-mediated colistin resistance in Gram-negative pathogens 157. Notably, we also investigated the structure-activity relationship of melatonin and revealed the importance of the indole moiety in its synergistic activity.

### Tet(X)-expressing pathogens

Tigecycline belongs to a new group of tetracyclines known as glycylcyclines. It has excellent oral availability and broad-spectrum antibacterial activity against both Gram-positive and Gram-negative bacteria 158. Notably, different from other tetracyclines, tigecycline can circumvent common tetracyclines resistance mechanisms involved in efflux pump and ribosomal protection 159. Thus, tigecycline is recognized as a last-option antibiotic against superbugs, particularly MDR Gram-negative bacteria.

**Figure 6:**
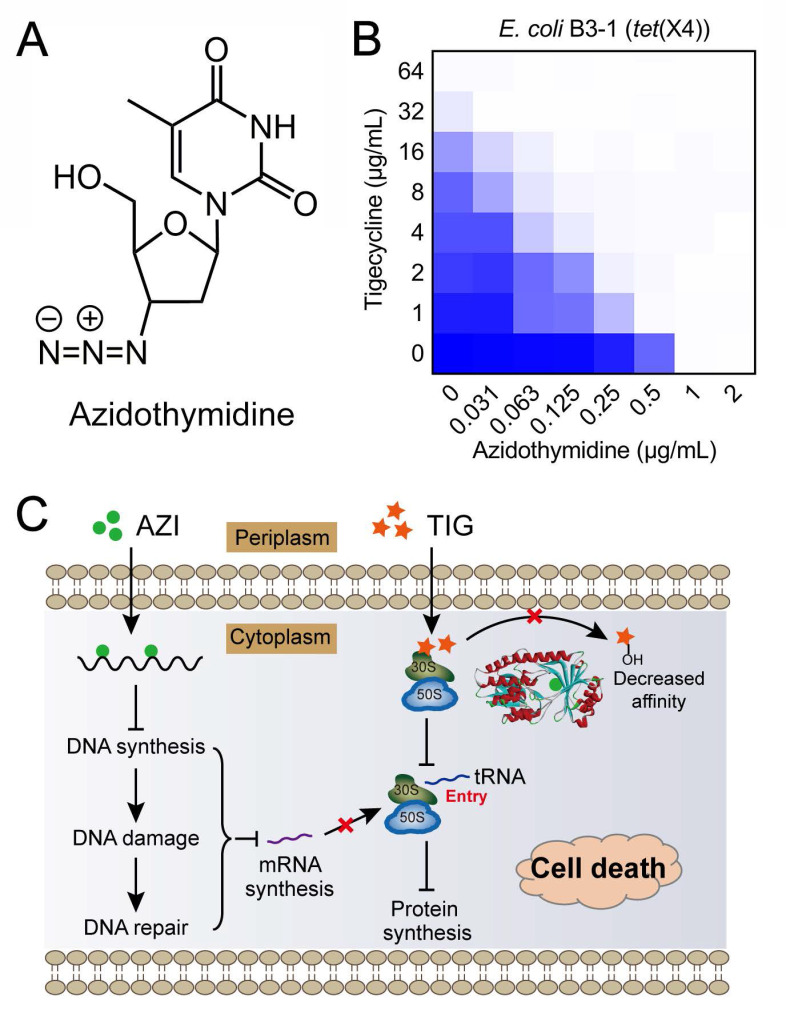
** Anti-HIV agent azidothymidine potentiates tigecycline activity against Tet(X)-expressing *E. coli*. (A)** Chemical structure of azidothymidine. **(B)** Checkerboard assay between tigecycline and azidothymidine against *tet*(X4)-positive *E. coli* B3-1. **(C)** Scheme of synergistic mechanisms of tigecycline in combination with azidothymidine. Adapted with permission from 162, Copyright 2020 Springer Nature.

As earlier described, *tet*(X) and its variant *tet*(X2) have been identified in *Bacteroides* species where they confer a low-level of tigecycline resistance. However, only a small proportion of *tet*(X)-positive bacteria have been reported, and non-plasmid-borne *tet*(X) genes exist. In 2019, two plasmid-mediated high-level tigecycline resistance genes,* tet*(X3) and *tet*(X4), were identified in numerous Enterobacterales and *Acinetobacter* 160, 161. Moreover, Tet(X3) and Tet(X4) inactivate all tetracyclines including the newly FDA-approved eravacycline and omadacycline, causing a remarkable MIC increase (64 to 128-fold). The identification of mobilized tigecycline resistance has seriously lowered its clinical efficacy against bacterial infections. To overcome *tet*(X3/X4)-mediated tigecycline resistance, our group conducted a cell-based screening from previously approved compounds. Further, we identified anti-HIV agent azidothymidine **(Figure 6A)** as a potent tigecycline adjuvant, which substantially decreased Tet(X)-mediated bacterial resistance to tigecycline in *tet*(X4)-positive *E. coli* **(Figure 6B)** 162. Additional experiments indicated that azidothymidine inhibited DNA synthesis, thus resulting in DNA damage and SOS response. In addition, azidothymidine can specifically bind to the Tet(X) catalytic pocket and block its enzymatic activity, thereby restoring the activity of tigecycline against Tet(X)-expressing bacteria **(Figure 6C)**. Interestingly, azidothymidine alone has been found to be effective on drug-resistant Gram-negative bacteria, including colistin or carbapenemase-resistant strains 163. However, the possibility of resistance development to azidothymidine by inactivation of thymidine kinase may impair its efficacy 164. The combination use of azidothymidine and other drugs such as the above mentioned tigecycline, provides an approach to circumvent the selection of resistant strains.

## Conclusions and future perspectives

The widespread use, misuse and overuse of antibiotics in the treatment of bacterial infections and in farming has resulted in a high evolutionary pressure on bacteria, thus accelerating the emergence of antibiotic resistance. These antibiotic-resistant bacteria are increasingly undermining existing anti-infective agents and, hence, constitute a global challenge in public health. Accordingly, there is an urgent and unmet need to identify novel therapeutic regimens. However, sustained high failure rates and costs in discovery of new antibiotics is a challenge, and spurs a growing interest in the use of combination therapies. The following three combinations: combination I (antibiotic + antibiotic); combination II (antibiotic + non-antibiotic); and combination III (non-antibiotic + non-antibiotic) afford promising pipelines for the discovery and development of new anti-infective regimens in the post-antibiotic era. Compared with monotherapy, antibiotic combination I can achieve a broad-spectrum coverage against pathogens particularly unknown or unidentified infective organisms. It also provides an empirical option for acute infections that need rapid treatment. However, this empirical medication can easily lead to negative outcomes, where excessive use may include many unapproved combinations. For instance, approximately 200 fixed-dose antibiotic combinations are available in India and only one third have been given regulatory approved 165. The lack of rapid, reliable diagnostics exacerbates the prescription of unnecessary or inappropriate antibiotic combination in clinics. Subsequently, this promotes antibiotic exposure and accelerates the emergence of resistance in the health-care setting.

Combination III represents an unexplored frontier that is currently accessible through more advanced technology such as computer-aided approaches 108. The immense landscape of genetic interactions has been exploited for drug combinations against drug-resistant bacteria. Since the chemical-genetic signatures are species-specific, this strategy may accelerate the development of narrow-spectrum drug combinations. Nevertheless, the therapeutic efficacy of the two non-antibiotic compounds *in vivo*, especially in the presence of complex body fluids, is still an unresolved concern.

Combination II (antibiotic adjuvant strategy) provides a promising strategy for the development of new therapeutic options. Currently, the discovery of novel antibiotics is extremely difficult and the combination II approach prolongs the life of well-established and clinically validated antibiotics. The outstanding success of antibiotic adjuvants is the inhibitor of serine-β-lactamases, which have been verified as effective in potentiating β-lactams activity and improving treatment outcome in clinical trials. In the case of antibiotic adjuvants, if new compounds are developed as adjuvants, then this cycle and investment is similar to the development of new antibiotics. In contrast, the screening of suitable antibiotic adjuvants from previously approved drugs cuts the development time, especially the clinical evaluation of safety. Undoubtedly, the reuse of drugs provides a reliable shortcut for the development of novel antibiotic adjuvants.

**Figure 7:**
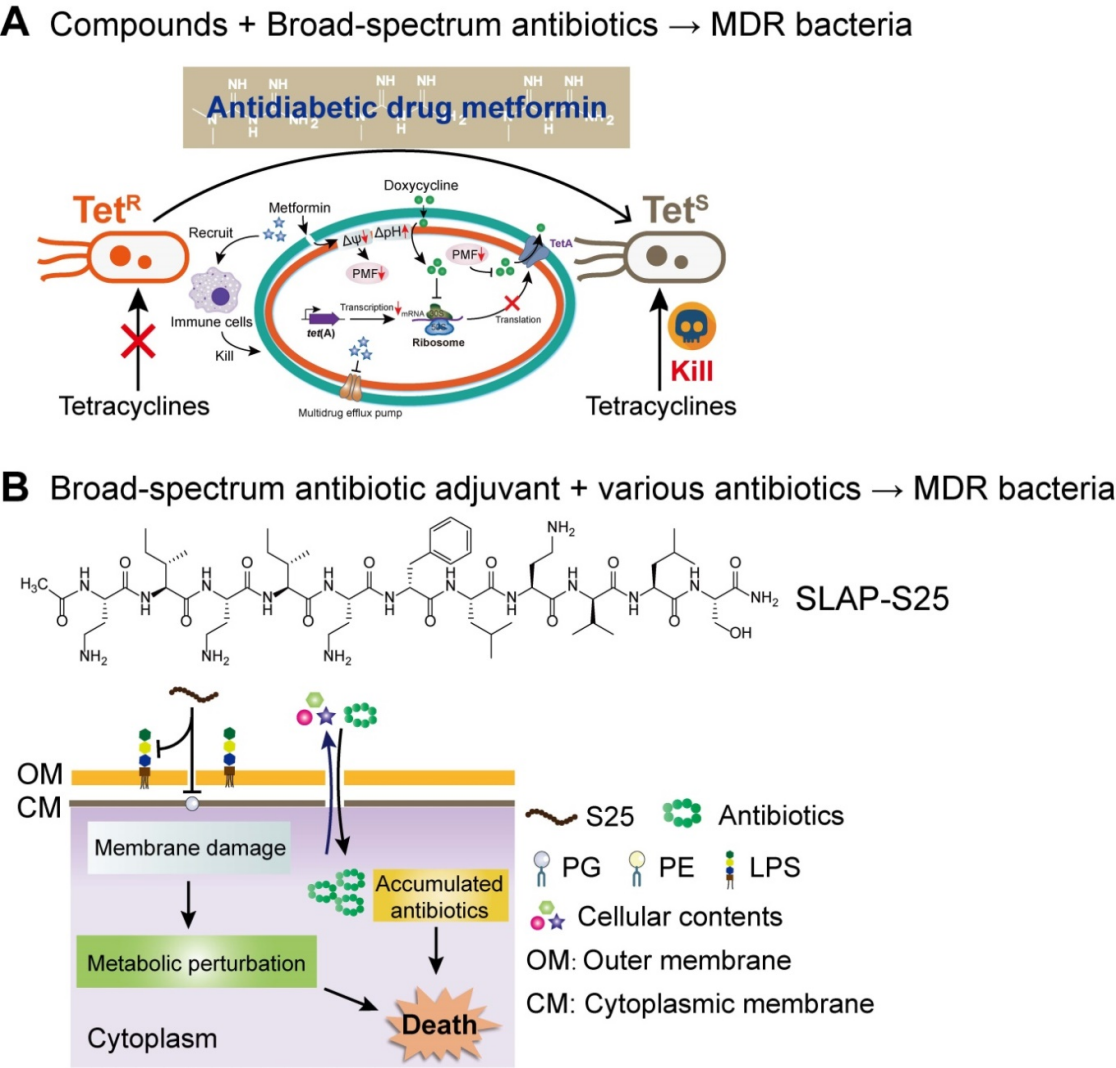
** Next-generation broad-spectrum combination therapies against MDR bacteria. (A)** Antidiabetic drug metformin restores broad-spectrum antibiotic tetracycline activity against MDR bacteria both *in vitro* and *in vivo* by promoting intracellular accumulation of antibiotics, as well as boosting the immune response and alleviating the inflammatory response. Adapted with permission from 166, Copyright 2020 John Wiley & Sons, Ltd. **(B)** SLAP-S25 boosts the activity of multi-classes of antibiotic against MDR Gram-negative bacteria by binding to LPS in the outer membrane (OM) and phosphatidylglycerol (PG) in the cytoplasmic membrane. Adapted with permission from 167, Copyright 2020 Springer Nature.

Compared with the developmental trend of narrow-spectrum antibiotics, broad-spectrum antibiotic combination is one of the development goals of the next-generation combination therapies. The two ways of achieving broad-spectrum antibacterial activity include, either the adjuvant acting on a broad-spectrum antibiotic or enhancing the activity of multiple antibiotics. Compared to earlier studies, our recent study about the synergistic activity between antidiabetic drug metformin and tetracyclines provided a great paradigm **(Figure 7A)**. Surprisingly, through cell-based screening, we found that metformin exhibited a great synergistic effect on tetracycline antibiotics, particularly doxycycline and minocycline, against a variety of MDR bacteria 166. Mechanistic experiments demonstrated that metformin promoted intracellular accumulation of doxycycline in tetracycline-resistant *E. coli*. In addition, metformin boosted the immune response and alleviated the inflammatory responses *in vitro*. As a proof-of-concept, metformin fully restored the activity of doxycycline in three animal infection models, implying the huge potential of metformin as a novel tetracycline adjuvant in combating MDR bacteria. With respect to the development of novel broad-spectrum adjuvants, the undecapeptide SLAP-S25 is an existing example **(Figure 7B)**. SLAP-S25 solely showed weak antibacterial activity but boosted the efficiency of all major classes of antibiotics against MDR Gram-negative pathogens 167. Mode-of-action studies revealed that SLAP-S25 triggered membrane damage by binding to both LPS in the OM and phosphatidylglycerol (PG) in the cytoplasmic membrane, thereby potentiating antibiotic efficacy through collaborative strategies. In addition, cranberry proanthocyanidin (cPAC) also displayed broad-spectrum potentiation to a range of antibiotics against various pathogenic Gram-negative bacteria both* in vitro* and *in vivo*, by enhancing membrane permeability and repressing multidrug efflux pumps 168.

As the saying goes, *while the prospects are bright, the road has twists and turns*, the same applies herein. The principal challenge for the successful deployment of combination therapies is whether the synergistic effect can be exerted *in vivo*. Specifically, this involves many key determinants, such as the complex pharmacology, pharmacokinetics and the dynamic of drugs *in vivo* 169, 170. This is mainly because drug-drug interactions are usually dose-dependent, thus improper combinations of antibiotics may lower the efficacy or even increase the risk of resistance. For instance, synergistic partners with discordant pharmacokinetic profiles display limited co-exposure to the target tissue, thus thwarting translation of *in vitro* activity to *in vivo* efficacy 69. Therefore, the determination of dosages in combinatorial drug therapy is critical to its clinical application. Besides, the synergistic activity of drug combinations may be accompanied by enhanced toxicity and side effects 171. Therefore, a systematic toxicological evaluation is required prior to clinical trials. Despite these challenges and obstacles, combination therapies, particularly antibiotic adjuvant options, undoubtedly are a new dawn for the treatment of infectious diseases in this era of antibiotic development depletion and bacterial resistance outbreaks. Furthermore, its feature of resistance development being slower than under monotherapy is compelling. Given the success of combination therapies in the current clinical setting, new combinations, especially antibiotic adjuvant strategies based on drug repurposing, have the most promising potential for improving the treatment of infectious diseases in the future.

## Figures and Tables

**Table 1:** Representative examples of drug repurposing as β-lactam adjuvants against MRSA

Compounds	Chemical structures	Clinical indications	Mechanisms of action	Refs
Zaragozic acid (lipid-lowering drugs)	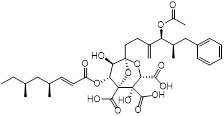	Hypercholesterolemia	Disassembles FMMs, disables PBP2a oligomerization and re-sensitize MRSA to penicillin	120
MAC-545496 (anti-virulence agent)	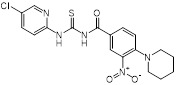	MRSA infections	GraR inhibitor, reverses β-lactam resistance in MRSA	121
Hypericin (anticancer and antidepressant agent)	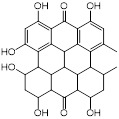	Nonmelanoma skin cancers	Inhibits *sarA* expression and enhances β-lactam activity against MRSA	125
Ticlopidine (platelet aggregation inhibitor)	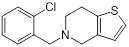	Circulatory disorders caused by high platelet aggregation	Wall teichoic acid biosynthesis inhibitor, synergizes with β-lactam against MRSA	126
Clomiphene (fertility drug)	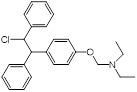	Human infertility	Undecaprenyl phosphate synthase inhibitor, synergizes with cell wall-targeted antibiotics against MRSA	127
Auranofin (TrxR inhibitor)	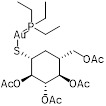	Rheumatoid arthritis	Synergizes with linezolid or fosfomycin against MRSA	128
Ebselen (Small molecule antioxidants)	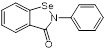	Meniere's disease and diabetes mellitus	Acts synergistically with traditional antimicrobials against MRSA	129

**Table 2:** Repurposing previously approved drugs as metallo-β-lactamase inhibitors

Mechanisms	Compounds	Clinical indications	Chemical structures	Refs
(1) Metal ion binding	Aspergillomarasmine A (angiotensin-converting enzyme inhibitor)	Hypertension	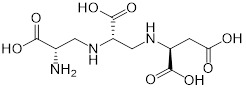	136
	Captopril (angiotensin-converting enzyme inhibitor)	Hypertension and congestive heart failure	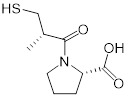	140
	Disulfiram (alcohol-abuse drug)	Chronic alcoholism	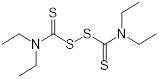	143
	Thiorphan (enkephalinase inhibitor)	Adult acute diarrhea	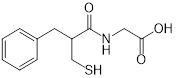	144
	Tiopronin (miscellaneous genitourinary tract agent)	Cystinuria	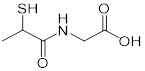	144
	Benzophenone (ultraviolet absorbents)	Metabolic diseases	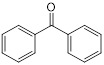	145
(2) Covalent bond formation	Cefaclor (antibiotic)	Bacterial infections	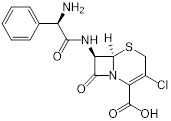	146
	Ebselen (voltage-dependent calcium channel (VDCC) inhibitor)	Meniere's disease and diabetes mellitus	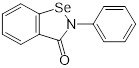	147
	Pterostilbene (natural dietary antioxidant)	Skin diseases	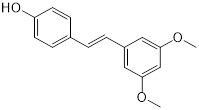	148
(3) Unknown	Mitoxantrone (topoisomerase II)	Acute leukemia, malignant lymphoma and cancers	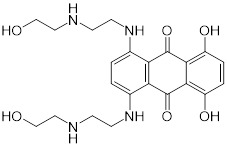	149

## Abbreviations

AMR: antimicrobial resistance
MRSA: methicillin-resistant *Staphylococcus aureus*
CRE: carbapenem-resistant Enterobacterales
MCRPE: MCR-producing Enterobacterales
PMF: proton motive force
ESBL: extended-spectrum β-lactamases
MDR: multidrug-resistant
OM: outer membrane
FDA: Food and Drug Administration
MBLs: metallo-β-lactamase
KPC: *K. pneumoniae* carbapenemase
NDM: New Delhi metallo-β-lactamase
VIM: verona integron-encoded metallo-β-lactamase
IMP: imipenemase metallo-β-lactamase
OXA-48: oxacillinase-48
PBP: penicillin-binding protein
LPS: lipopolysaccharides
MIC: minimum inhibitory concentration
FICI: fractional inhibitory concentration index
PG: phosphatidylglycerol
